# Rapid Determination of Cellulose and Hemicellulose Contents in Corn Stover Using Near-Infrared Spectroscopy Combined with Wavelength Selection

**DOI:** 10.3390/molecules27113373

**Published:** 2022-05-24

**Authors:** Na Wang, Jinrui Feng, Longwei Li, Jinming Liu, Yong Sun

**Affiliations:** 1 College of Information and Electrical Engineering, Heilongjiang Bayi Agricultural University, Daqing 163319, China; yaya588588@163.com (N.W.); 18945521643@163.com (J.F.); longxiaowei1988@163.com (L.L.); 2 National Coarse Cereals Engineering Research Center, Daqing 163319, China; 3 College of Engineering, Northeast Agricultural University, Harbin 150030, China

**Keywords:** near-infrared spectroscopy, cellulose and hemicellulose contents, backward partial least squares, competitive adaptive reweighted sampling, genetic simulated annealing algorithm

## Abstract

The contents of cellulose and hemicellulose (C and H) in corn stover (CS) have an important influence on its biochemical transformation and utilization. To rapidly detect the C and H contents in CS by near-infrared spectroscopy (NIRS), the characteristic wavelength selection algorithms of backward partial least squares (BIPLS), competitive adaptive reweighted sampling (CARS), BIPLS combined with CARS, BIPLS combined with a genetic simulated annealing algorithm (GSA), and CARS combined with a GSA were used to select the wavelength variables (WVs) for C and H, and the corresponding regression correction models were established. The results showed that five wavelength selection algorithms could effectively eliminate irrelevant redundant WVs, and their modeling performance was significantly superior to that of the full spectrum. Through comparison and analysis, it was found that CARS combined with GSA had the best comprehensive performance; the predictive root mean squared errors of the C and H regression model were 0.786% and 0.893%, and the residual predictive deviations were 3.815 and 12.435, respectively. The wavelength selection algorithm could effectively improve the accuracy of the quantitative analysis of C and H contents in CS by NIRS, providing theoretical support for the research and development of related online detection equipment.

## 1. Introduction

With the development of society, the demand for energy sources such as coal, oil, and natural gas is increasing [1]. However, the consumption of a large amount of limited fossil energy will inevitably lead to the problem of energy shortage, accompanied by an increase in climate change, environmental pollution, and other problems [2]. Finding new and clean renewable energy sources is an important research direction aimed at achieving high-quality development in energy use [3]. Biomass is an example of a type of green renewable energy source, and agricultural straw is one of the biomass resources with the most potential for high-value applications [4]. Corn stover (CS) is one of the three primary straw resources in China, and its main components are cellulose, hemicellulose, and lignin [5]. Anaerobic fermentation is one of the available ways of realizing the resource utilization of straw, and its advantages are mainly low cost and lower secondary pollutant production [6]. The cellulose and hemicellulose (C and H) contents of CS directly affect the methane yield in anaerobic fermentation. In order to effectively control the anaerobic fermentation process of CS, the contents of C and H in CS should be measured accurately and quickly [7]. However, traditional chemical methods have the disadvantages of being time-consuming and labor-intensive, with high-cost, and these traditional methods are not suitable for rapid and efficient quantitative determination of an enormous number of samples.

Near-infrared spectroscopy (NIRS) has the advantages of enabling rapid, simple, and nondestructive analyses, and it has been widely used in quantitative analysis (QA) and qualitative analysis of agricultural products and wastes [8]. NIRS mainly records the frequency overtone and absorption combination of a hydrogen-containing group’s vibrations [9]. The absorption wavelengths and intensities for different groups or for the same group are different in different chemical environments. Therefore, NIRS is well suited to measuring the composition of hydrocarbon organic substances [10]. However, there are some problems regarding NIRS, such as wide peak width, serious overlapping, and poor spectral interpretation, and therefore it is necessary to use chemometrics methods for QA [11]. Cellulose and hemicellulose contain many hydrogen-containing groups (C–H, –OH, etc.), which are suitable for constructing qualitative and quantitative detection models using NIRS combined with chemometrics [12].

With developments in science and technology, the acquisition accuracy of near-infrared spectrometers is increasing. If the correction model is established directly with the wavelength variables (WVs) of the whole spectrum, the accuracy and robustness of the model will eventually be affected, due to the weak correlation between some spectral WVs and the components [13]. To effectively extract the characteristic WVs (CWVs) with high correlation and to establish a simpler and more stable NIRS model, scholars have proposed using interval partial least squares [14], synergy partial least squares [15], backward partial least squares (BIPLS) [16], and other spectral area optimization algorithms, together with uninformative variable elimination [17], competitive adaptive weighted sampling (CARS) [18], and various other wavelength selection algorithms, and genetic algorithms (GA) [19], genetic simulated annealing algorithms (GSA) [20], ant colony algorithms [21], particle swarm optimization algorithms [22], and various other intelligent optimization algorithms to effectively filter out WVs. Sometimes a single WV optimization method fails to meet the requirements of the analysis, and a combination of methods is required [23,24,25]. BIPLS, CARS, and GSA are the most typical methods used in spectral region optimization, wavelength selection, and intelligent optimization, respectively, and are widely used in wavelength selection for high-dimensional spectral data [26,27,28].

In this paper, the methods of BIPLS, CARS, and GSA were combined to select the CWVs for C and H in CS, and a quantitative calibration model was established for the chemical contents of the main components in CS and their near-infrared spectral data. By comparing the predictive performances of different methods, rapid detection and analysis for C and H contents in CS could be realized.

## 2. Results and Discussion

### 2.1. Spectral Data Analysis

**Figure 1:**
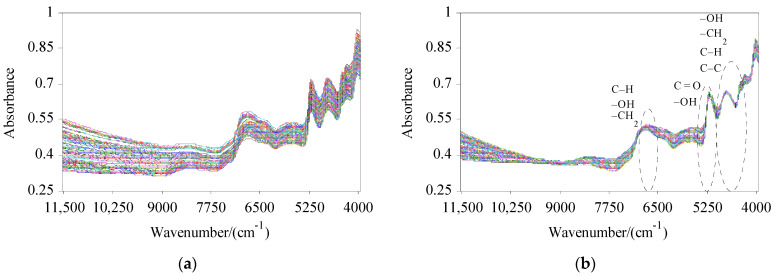
Near-infrared reflectance spectra of all samples: (**a**) raw spectra of all samples; (**b**) preprocessed spectra of all samples.

The NIRS data collected by spectral scanning of 184 CS samples are shown in Figure 1a. The trend of each spectral line in the original spectra is roughly similar, and the spectral line distribution is relatively broad. By comparing the RMSECV values of the PLS model established by SNV, MSC, FD, SG, normalization, and their combinations, the preprocessing method was determined to be SG + MSC, according to the principle of minimizing the RMSECV. The preprocessing modifies the random high-frequency noise and scattering problems in the spectra (Figure 1b) [29]. There are multiple absorption peaks at 6817 cm^−1^, 5182 cm^−1^, 4749 cm^−1^, and 4292 cm^−1^ in the spectral region, which can reflect information on different components of the samples and provide a great deal of information for the QA.

In the whole spectra, different samples have similar absorption peaks, indicating that the main components contained in the samples were similar, while the intensities of the absorption peaks were different due to the differences in the contents of components in the samples. Near 6817 cm^−1^ is the characteristic absorption band of second-order frequency multiplication of C–H, –OH, and –CH_2_ groups. Near 5182 cm^−1^ is the first-order frequency multiplication band of C=O and –OH groups. Near 4749 cm^−1^ and 4292 cm^−1^ are the first combination frequency bands of –OH, C–C, C–H, and –CH_2_ groups. By selecting the WVs near the wave crest, the prediction model for C and H in CS can be well established [10].

**Figure 2:**
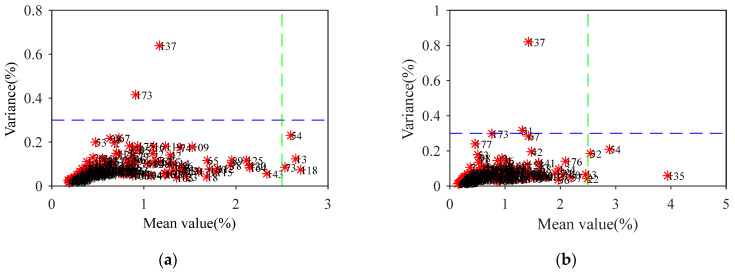
The predicted residual mean and variance distribution map for cellulose (**a**) and hemicellulose (**b**).

The MCCV algorithm [30] was run 1000 times on the data for the 184 straw samples, and a predicted residual mean and variance distribution map was drawn for each sample (Figure 2). For cellulose, the RMSECV value of the PLS model was reduced to 0.862 by eliminating 6 samples with a mean value greater than 2.5 or a variance greater than 0.3: No. 13, No. 54, No. 73, No. 118, No. 137, and No. 173. As a result, the RMSECV was reduced by 0.143. Following the same method, 6 samples were removed as abnormal samples of hemicellulose: No. 22, No. 31, No. 32, No. 54, No. 135, and No. 137.

Using a random selection (RS) algorithm, 46 samples from the 178 sample sets were randomly selected as the ITset. Using SPXY, 132 samples were split in a ratio of 2 to 1 into the Cset and Vset. The content distributions of C and H in the Cset and Vset are shown in Table 1. The ranges of cellulose contents in the Cset and Vset were 36.067–51.527 and 37.440–49.080, respectively, and the ranges of hemicellulose contents in the Cset and Vset were 9.484–38.541 and 10.245–38.388, respectively. The sample component content of Cset covered the sample component content of Vset, which shows that the distribution of the sample set divided by SPXY is reasonable, and the model established in this way can better predict unknown samples [31]. The minimum content of cellulose in the ITset was less than the minimum value for Cset, and the maximum content of hemicellulose was greater than the maximum value for Cset, resulting in a good test of the robustness of the regression model [9].

### 2.2. Wavelength Variables Selection

#### 2.2.1. BIPLS-Selected Characteristic Sub-Intervals

The full spectral data were divided into *n* sub-intervals (*n*= 61, 46, 36, 26, 18, and 12) representing about 30, 40, 50, 70, 100, and 150 WVs, respectively, for the BIPLS characteristic spectral region optimization. As shown in Table 2, when *n* = 46, the RMSECV corresponding to cellulose was the smallest. When *n* = 61, the RMSECV value was slightly higher than for *n* = 46, so it was speculated that the number of optimal interval divisions for cellulose was between 46 and 61. In the same way, it was speculated that the number of optimal interval divisions for hemicellulose was between 26 and 36. To further determine the optimal *n* value for C and H, the BIPLS algorithm was run adding 1 successively in the cellulose (46–61) and hemicellulose (26–36) regions, respectively, to obtain the optimal characteristic sub-interval of BIPLS. The optimal *n* value of cellulose was 47, and the corresponding RMSECV was the smallest (0.676). BIPLS optimally selected 11 characteristic spectral regions for cellulose (5, 6, 12, 19, 22, 26, 30, 35, 40, 43, and 47) and 432 CWVs. In the same way, the optimal *n* value of hemicellulose was 30, and 5 characteristic spectral regions (13, 22, 25, 27, and 29) and 306 CWVs were selected. It can be seen from Table 2 that it is crucial to select an appropriate *n* value when using the BIPLS algorithm to optimize the characteristic wavelength [32].

#### 2.2.2. CARS-Selected Characteristic Wavelength Variables

**Figure 3:**
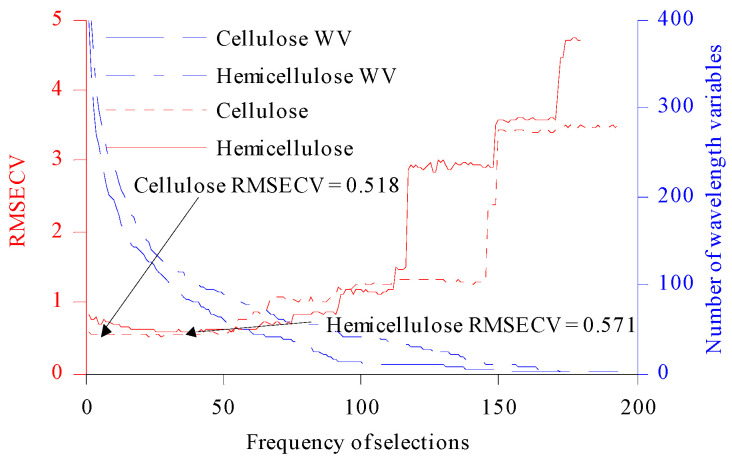
The relationship between RMSECV and WV and the number of selections. WV: wavelength variable.

When we used CARS to optimize the CWVs for C and H, the number of MCSs was firstly set to 1000, and the PLS model was established by taking 80% of the samples in the Cset. Then, MCS and ARS were combined to compete in selecting CWVs. The wavelength subset was set to 100, and the minimum RMSECV in the selected subset was that for the CWVs optimized by CARS. Two random variables, MCS and ARS, led to uncertainty in the results of each CARS optimization. The CARS algorithm was executed 200 times in the experiment (denoted CARS200). As shown in Figure 3, the RMSECV values for C and H first decreased slowly with an increase in the number of repeated selection times, then showed an overall upward trend, and finally tended to be flat.

According to the minimum RMSECV (cellulose: 0.518; hemicellulose: 0.571), the corresponding CWVs were selected [7]. A total of 241 CWVs were selected for cellulose, and 106 CWVs were selected for hemicellulose. In the third-order overtone band range of the –OH group, 98 CWVs were selected for cellulose and 32 CWVs were selected for hemicellulose. In the second-order overtone band range of C–H, and –CH_2_ groups, 63 CWVs were selected for cellulose and 32 CWVs were selected for hemicellulose. In the range of the first-order overtone band range of C=O and –OH groups, 30 CWVs were selected for cellulose and 13 CWVs were selected for hemicellulose. In the combined frequency range of C=O, –OH and C–C groups, 50 CWVs were selected for cellulose and 29 CWVs were selected for hemicellulose [33].

#### 2.2.3. BIPLS-CARS-Selected Characteristic Wavelength Variables

**Figure 4:**
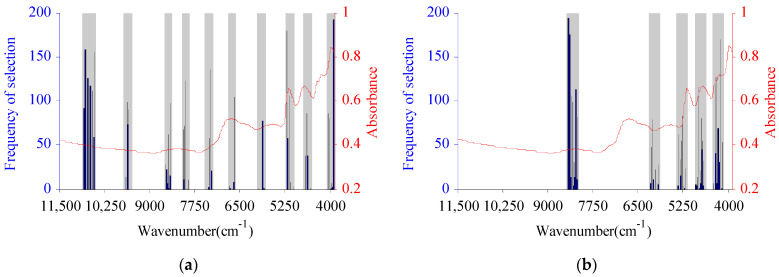
The characteristic wavelength variables selected by BIPLS-CARS optimized for cellulose (**a**) and hemicellulose (**b**).

Since the selection of CWVs by the BIPLS algorithm is based on the characteristic intervals, there may still be redundant data in the band selection process. To further remove redundant information variables, CARS was used to optimize the WVs in the characteristic spectral region optimized by BIPLS [24]. The CARS algorithm was executed 200 times. According to the principle of taking the smallest RMSECV, the numbers of CWVs for C and H were 169 and 115, respectively, i.e., 263 and 191 fewer than those of BIPLS alone. The most frequently selected CWV of BIPLS-CARS for cellulose was 5227 cm^−1^, selected 180 times, which was located in the first-order overtone range of –OH and C=O groups. The WVs most frequently selected by BIPLS-CARS for cellulose were basically located in the 9622–10,854 cm^−1^, 80,368,531 cm^−1^, 7332–7381 cm^−1^, 6677–6697 cm^−1^, 5157–5227 cm^−1^, and 4663–4712 cm^−1^ ranges. Among these, 9622–10,854 cm^−1^ was in the third-order overtone band range of –OH groups, 8036–8531 cm^−1^ was in the second-order overtone band range of C–H and –CH_2_ groups, 7332–7381 cm^−1^ was in the second-order overtone band range of –OH groups, 6677–6697 cm^−1^ was in the first-order overtone band range of C=O groups, 5157–5227 cm^−1^ was in the first-order overtone band range of C=O and –OH groups, and 4663–4712 cm^−1^ was located in the first-order overtone band range of C=O and the combination frequency range of C–C groups. The CWV most frequently selected by BIPLS-CARS for hemicellulose was 8473 cm^−1^, selected 194 times, which was located in the second-order overtone band range of C–H and –CH_2_ groups. The WVs most frequently selected by BIPLS for hemicellulose were basically located in the 8226–8477 cm^−1^, 5243–5429 cm^−1^, 4728–4885 cm^−1^, and 4197–4415 cm^−1^ ranges. Among these, 8226–8477 cm^−1^ was in the second-order overtone band range of C–H and –CH_2_ groups, 5243–5429 cm^−1^ was in the first-order overtone band range of C=O groups, 4728–4885 cm^−1^ was in the combination band range of C=O and –OH groups, and 4197–4415 cm^−1^ was in the combination band range of C–H and –CH_2_ groups [34]. The CWVs of C and H selected by BIPLS-CARS optimized for C and H are shown in Figure 4.

#### 2.2.4. BIPLS-GSA- and CARS-GSA-Selected Characteristic Wavelength Variables

When using GSA to re-optimize the WVs selected by BIPLS and CARS, the optimal results of BIPLS (cellulose: 432; hemicellulose: 306) and those of CARS (cellulose: 241; hemicellulose: 106) were used as the code lengths for reselection. The GSA’s optimum parameters for C and H were an initial temperature of 200, a cooling coefficient of 0.90, and a maximum number of genetic generations of 200. The number of repeated selections when the RMSECV was the smallest was used as the threshold. The WVs whose number of selections exceeded this threshold were used as the CWVs selected by the GSA. After BIPLS and CARS were optimized by the GSA, the WVs for C and H were 241 and 138 for BIPLS-GSA and 200 and 70 for CARS-GSA, respectively.

#### 2.2.5. Comparison of Optimized Results

**Figure 5:**
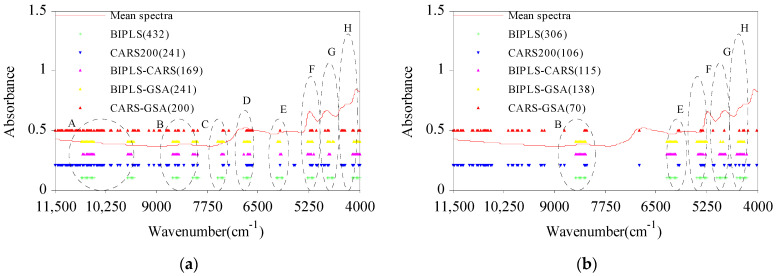
The characteristic wavelength variable distributions of cellulose (**a**) and hemicellulose (**b**) optimized by BIPLS, CARS200, BIPLS-CARS, BIPLS-GSA, and CARS-GSA.

Compared with the full spectral model (denoted Full-PLS), the five CWV selection algorithms effectively reduced the number of wavelengths (Figure 5). Among these, BIPLS had the largest numbers of WVs at 432 for cellulose and 306 for hemicellulose, accounting for 23.415% and 16.585% of the full spectral wavelength range, respectively. BIPLS-CARS (cellulose 169) and CARS-GSA (hemicellulose 70) had the lowest numbers of WVs for C and H, accounting for 9.160% and 3.794% of the full spectral wavelength range, respectively. Compared with BIPLS, the optimized WVs of BIPLS-CARS were obviously reduced (cellulose: 60.880%; hemicellulose: 62.418%). The numbers of WVs optimized by BIPLS-GSA were less than the numbers for BIPLS but slightly higher than the numbers for BIPLS-CARS. The numbers of WVs for C and H optimized by CARS-GSA were reduced by about 17.012% and 33.962%, respectively, compared with CARS. Among several wavelength selection algorithms, the WVs selected by BIPLS, BIPLS-CARS, and BIPLS-GSA were relatively concentrated, and the WVs selected by CARS200 and CARS-GSA were relatively uniform. Region A corresponds to the third-order overtone band range of –OH groups, regions B and C correspond to the second-order overtone band range of C–H, –CH_2_, and –OH, respectively, regions D, E, and F correspond to the first-order overtone band range of C=O, C–H, and –OH, and regions G and H correspond to the combination frequency of C–C, C–H, and –CH_2_. When the five algorithms were used to optimize the characteristic wavelengths of cellulose, some CWVs in the A, B, C, D, E, F, G, and H regions were selected. When the five algorithms were used to optimize the characteristic wavelengths of hemicellulose, some CWVs in the B, E, F, G, and H regions were selected.

### 2.3. Analysis of Regression Models

The regression models of Full-PLS, BIPLS, CARS200, BIPLS-CARS, BIPLS-GSA, and CARS-GSA for C and H were established, and the modeling accuracies of the six models were compared (Table 3).

The Full-PLS data set is large, the modeling and prediction processes take a long time, and the equipment performance requirements are high. It can be seen from Table 3 that the five optimal algorithms eliminate a large number of WVs unrelated to the contents of C and H. In the process of optimization, the time taken to establish the prediction model for Full-PLS was the shortest, at 14.043 min for cellulose and 15.358 min for hemicellulose; BIPLS-GSA took the longest time, at 1858.209 and 1801.827 min for cellulose and hemicellulose, respectively. The modeling time is related to the algorithm used and the number of runs. The results of models optimized by different algorithms were different, but the performance of PLSRM after wavelength selection was better than that of Full-PLS, which further proves the importance of CWV selection in the full spectra. The amounts of spectral data used for modeling were significantly reduced after wavelength selection. After wavelength selection, the amounts of spectral data used for modeling were significantly reduced, and the times required to predict new samples using the optimized model were reduced. The minimum RPDs for C and H were 3.448 and 10.529, and these RPDs are greater than 3. It is generally believed that when the relative RMSE is less than 5%, the model can meet the needs of actual chemical analysis [9]. The largest relative RMSEs for cellulose and hemicellulose in the model were 1.914% and 3.999, respectively, and these values are less than 5%. The results show that the QA model established using NIRS can meet the requirements for measuring the C and H contents in CS [20].

The RMSEP values of the regression models for cellulose established by the CWVs optimized by BIPLS and CARS decreased from 0.870 to 0.830 and 0.861, and the RPD values increased from 3.448 to 3.612 and 3.482, respectively, compared with Full-PLS. The RMSEP values of the regression models for cellulose established by the WVs optimized by BIPLS and CARS decreased from 1.033 to 0.927 and 0.922, and the RPD values increased from 10.529 to 11.982 and 12.041, respectively, compared with Full-PLS. The number of WVs optimized by CARS was significantly less than the number optimized by BIPLS, but there was a problem of performance instability.

In BIPLS-CARS and BIPLS-GAS, BIPLS was used to select the effective characteristic spectral region, and CARS and GSA were used to select the relevant CWVs from the characteristic spectral region. BIPLS-CARS eliminated the most redundant WVs of BIPLS (cellulose: 263; hemicellulose: 191). Compared with BIPLS, the RMSEP and RPD indicators of BIPLS-CARS were better. Compared with BIPLS-CARS, the number of WVs optimized by BIPLS-GSA was slightly higher, the RMSEC was marginally worse, and other performance indicators were higher than those of the BIPLS-CARS model. Among the five algorithms, the comprehensive performance index of the model established by CARS-GSA was the best.

In summary, BIPLS and CARS can effectively extract CWVs, but the effect is not obvious when they are used alone. BIPLS can obtain better model performance parameters when it used alone, but the number of WVs selected is large, which affects the calculation speed of the model. CARS dramatically reduces the number of WVs, and the distribution of WVs is relatively uniform. However, due to its randomness, CARS must be run multiple times to reduce the instability of the model. Combining the algorithms for WV optimization can effectively improve the model’s performance [35]. The performance indicators for the BIPLS-CARS, BIPLS-GSA, and CARS-GSA models were better than those of Full-PLS, BIPLS, and CARS. When CARS-GSA optimizes the CWV, it selects the discrete WVs with high effectiveness, and the model established by the preferred CWVs has the best performance [28].

**Figure 6:**
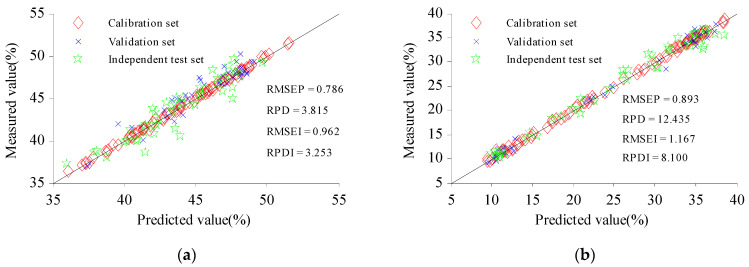
Prediction scatter plot for cellulose (**a**) and hemicellulose (**b**). RMSEI and RPDI represent the RMSE and RPD of the independent test set, respectively.

To further show the accuracy of the QA model for C and H, the ITset was added for verification. The predicted and measured values of the Cset and Vset of the samples were evenly distributed around the 1:1 line (Figure 6), which shows that the predicted value and the measured value have good fitting accuracy [36]. The ITset can better detect the robustness of the model [37]. The scatter of predicted values of the samples from the ITset had a low degree of dispersion near the 1:1 line. The RPD of the ITset for cellulose was 3.253, and the RPD of the ITset for hemicellulose was 8.100, indicating that CARS-GSA could accurately extract CWVs with high correlation for C and H, simplify the structure of the prediction model, and establish the NIRS rapid detection model, to meet actual requirements for the DA of C and H in CS [38].

## 3. Materials and Methods

### 3.1. Sample Collection and Processing

**Figure 7:**
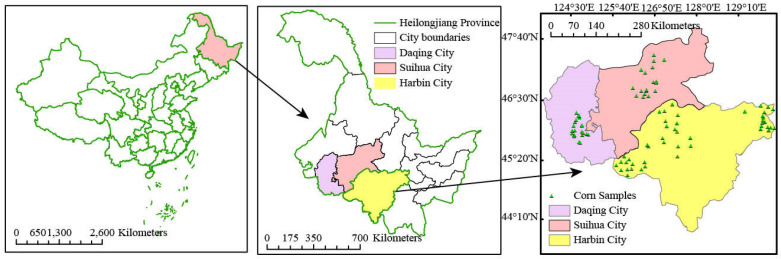
Distribution of the sampling locations.

CS samples were collected from Daqing, Harbin, and Suihua, Heilongjiang Province, China. The distribution of the sampling locations is shown in Figure 7. Surface impurities were removed from the collected CS with distilled water, and the samples were placed in an open and ventilated place for natural air-drying and then mechanically pulverized. After drying at a constant temperature in a drying oven at 40 °C for 48 h, all samples were pulverized using an FZ102 mill (Taisite, Tianjin, China), filtered through a 40 mesh vibrating screen, and marked and saved in sealed bags. A total of 184 samples were collected. The contents of C and H were measured using the Van Soest method [39]. An ANKOM 200i fiber analyzer (ANKOM Tech., New York, NY, USA) was used to measure the contents of neutral detergent fiber (NDF) and acid detergent fiber (ADF) in the sample powder [40]. The content of acid detergent lignin (ADL) was measured using the 72% sulfuric acid hydrolysis method [41]. The calculation methods for the C and H contents used NDF and ADF, and ADF and ADL, respectively.

### 3.2. Acquisition of Spectral Data

Spectral data were obtained using a TANGO near-infrared spectrometer (Bruker Optik, Ettlingen, Germany). First, the spectrometer was preheated 2 h in advance to ensure that the instrument worked stably. The measuring platform was set to rotating mode, and the measuring mode was set to integrating sphere diffuse reflection. The background was measured once per hour, the instrument’s resolution was 8 cm^−1^, the spectral range was 3940–11,542 cm^−1^, and scanning times were set to 32. The sample was placed into the sample cup at a thickness of about 1.5 cm, to cover the bottom of the cup fully. The indoor temperature and humidity were kept unchanged.

### 3.3. Optimization Method of Wavelength Variables

#### 3.3.1. BIPLS Algorithm

The BIPLS algorithm divides the whole spectrum into *n* equal-width sub-intervals, eliminates the interval with the worst correlation among the *n* intervals, performs partial least squares (PLS) regression on all the remaining sub-intervals, and calculates the corresponding root mean squared error of cross-validation (RMSECV) [16]. Then, the interval with the worst correlation among the *n* − 1 intervals is eliminated, the remaining *n* − 2 intervals are used to perform PLS regression, and the RMSECV is calculated again. The excluded sub-interval is the one with the worst performance among all the sub-intervals of the regression model and the one with the smallest model evaluation RMSECV after elimination. The process continues until only one sub-interval remains. The optimal characteristic spectral region is the combination of sub-intervals corresponding to the minimum RMSECV of each PLS model.

#### 3.3.2. CARS Algorithm

The CARS algorithm firstly establishes the PLS regression model (PLSRM) by Monte Carlo sampling (MCS), and then selects the WV with the largest absolute weight of regression coefficients in the PLS calibration model, based on adaptive re-weighted sampling (ARS) and an exponentially decreasing function (EDF), obtaining multiple WV subsets [18]. Then, a cross-validation model is established for each WV subset. Finally, the CWV selected by CARS is selected according to the minimum RMSECV value principle. Because of the randomness of ARS and the EDF, the results are different each time. To solve this problem, we executed the CARS algorithm multiple times and selected the multiple selected WVs as the final CWVs, according to the minimum RMSECV.

#### 3.3.3. GSA Algorithm

The GSA algorithm integrates the annealing strategy of the simulated annealing algorithm into the fitness function design of the GA and realizes the selection and replication of the perturbation solution in the GA evolution process using the Metropolis criterion [42]. GSA effectively solves the GA algorithm’s two problems of early maturity and low search efficiency in the later stage. To solve the problem of solution space divergence when GSA encodes with the whole spectral wavelength range as the code length, GSA was combined with BIPLS and CARS, respectively. The model’s prediction performance can be improved further by selecting high-correlation WVs from BIPLS and CARS to participate in the modeling. Due to the randomness of the GSA optimization results, a calibration model with good robustness and strong predictive ability was obtained by executing the GSA algorithm multiple times.

### 3.4. Model Construction and Evaluation

When establishing a fast PLS detection model for C and H contents in CS, the spectral data of the samples should first be preprocessed, and then the abnormal samples should be screened by the Monte Carlo cross-validation (MCCV) algorithm. To investigate the robustness of the QA model, an independent test set (ITset) was randomly selected to use for external validation. According to the distribution of the chemical component contents in the samples and the spatial distribution of principal component scores in the spectral data, the data were divided into a calibration set (Cset) and a validation set (Vset) using the sample set partitioning based on joint x-y distances (SPXY) method [43]. Using a reasonable number of latent variables (LVs) not only avoids overfitting of the model but also ensures that the model has better interpretation ability. The optimal number of LVs was selected by MCCV combined with the prediction residual error sum of squares (PRESS) of PLSRM [10]. By comparing the PRESS, the number of LVs with the lowest PRESS value was selected as the optimal number of LVs.

In this paper, the performance of the PLSRM established by the whole spectra was compared with the performances established by five methods: BIPLS, CARS, BIPLS combined with CARS (denoted BIPLS-CARS), BIPLS combined with GSA (denoted BIPLS-GSA), and CARS combined with GSA (denoted CARS-GAS). The statistical parameters of the modeling performance included the determination coefficient $R^{2}$, the root mean square error (RMSE), and the residual predictive deviation (RPD). $R^{2}$ represents the correlation between the predicted value and the actual value, and the closer $R^{2}$ is to 1, the better the stability of the model and the higher the fitting degree. When the value is greater than 0.9, it is considered that the prediction model meets the actual detection needs [28]. The RMSE represents the deviation between the predicted value and the actual value, and the closer the RMSE is to 0, the stronger the predictive ability of the model. The RPD is the standard deviation of the Vset divided by the RMSE of the Vset, which reflects the resolution and robustness of the model. When RPD ≥ 3, it is generally considered that the model has good predictive ability [44]. The formulas for $R^{2}$, RMSE, and RPD are as follows:

(1) $$R^{2}=1-\sum_{i=1}^{n}{\left(y_{i}-{\hat{y}}_{i}\right)}^{2}/\sum_{i=1}^{n}{\left(y_{i}-\bar{y}\right)}^{2}$$

(2) $$\mathrm{RMSE}=\sqrt{\sum_{i=1}^{n}{\left(y_{i}-{\hat{y}}_{i}\right)}^{2}/n}$$

(3) $$\mathrm{RPD}=\sqrt{\sum_{i=1}^{n}{\left(y_{i}-\bar{y}\right)}^{2}/\sum_{i=1}^{n}{\left(y_{i}-{\hat{y}}_{i}\right)}^{2}}$$

where $y_{i}$ is the measured value of the *i*-th sample, ${\hat{y}}_{i}$ represents the predicted value of the *i*-th sample, $\bar{y}$ is the mean of the measured values of all samples, and *n* is the number of samples.

In this study, all the algorithm processes (including identifying abnormal samples, the selection of characteristic spectral regions and characteristic wavelengths, and the construction of the PLSRM, etc.) were performed in the MATLAB R2012b software platform. The computer used to run the program was configured with an Intel (R) core (TM) i7–4790 processor, with a 3.6 GHz main frequency and 8 GB of memory.

## 4. Conclusions

This paper systematically introduced the use of BIPLS, CARS200, BIPLS-CARS, BIPLS-GSA, and CARS-GSA algorithms combined with chemometrics to select the CWVs of C and H in CS. It compared the performances of the PLSRMs established by the optimization results of each algorithm. By comparing comprehensive indicators, CARS-GSA was found to be the optimal method for determining the CWVs for C and H, among the five algorithms. The RMSEP values of the C and H models optimized by CARS-GSA were 0.786 and 0.893, respectively, i.e., 9.66% and 13.55% lower than the values of Full-PLS, and the RPD values were 3.815 and 12.435, respectively, i.e., 0.367 and 1.906 higher than the values of Full-PLS. The results show that wavelength selection can simplify the structure of the model and improve the performance. The CARS-GSA wavelength selection method can be used for constructing a NIRS rapid detection model for C and H contents in CS.

## Figures and Tables

**Table 1:** Content distribution of sample set.

Sample	Composition	Amount	Mean (%)	Max (%)	Min (%)	SD (%)
Cset	Cellulose	88	44.247	51.527	36.067	3.758
	Hemicellulose	88	23.760	38.541	9.484	9.828
Vset	Cellulose	44	45.433	49.080	37.440	3.034
	Hemicellulose	44	25.832	38.388	10.245	10.999
ITset	Cellulose	46	43.813	49.757	36.031	3.163
	Hemicellulose	46	25.123	38.592	9.948	9.554

Cset: calibration set; Vset: validation set; ITset: independent test set; SD: standard deviation.

**Table 2:** Preliminary selection results of spectral characteristic intervals for cellulose and hemicellulose, optimized using BiPLS.

Intervals	Cellulose			Hemicellulose		
	Selected Intervals	RMSECV (%)	Selected Wavelengths	Selected Intervals	RMSECV (%)	Selected Wavelengths
61	15	0.697	456	17	1.021	512
46	14	0.681	563	13	0.995	520
36	12	0.714	617	17	0.896	870
26	9	0.757	638	8	0.957	568
18	11	0.719	1128	8	1.130	819
12	9	0.747	1384	6	1.143	921

**Table 3:** The results for wavelength selection.

Component	Model	NW ^1^	LVs	$R_{c}^{2}$	$R_{p}^{2}$	RMSEC (%)	RMSEP (%)	RPD	MT ^2^ (m)	TT ^3^ (s)
Cellulose	Full-PLS	1845	15	0.980	0.917	0.527	0.870	3.448	14.043	1.598
	BIPLS	432	13	0.982	0.925	0.496	0.830	3.612	166.072	1.567
	CARS200	241	16	0.994	0.920	0.284	0.861	3.482	264.298	1.459
	BIPLS-CARS	169	10	0.977	0.928	0.565	0.802	3.738	367.505	1.427
	BIPLS-GSA	241	11	0.979	0.927	0.541	0.801	3.747	1858.209	1.450
	CARS-GSA	200	8	0.971	0.930	0.628	0.786	3.815	1523.729	1.433
Hemicellulose	Full-PLS	1845	18	0.998	0.990	0.383	1.033	10.529	15.358	1.638
	BIPLS	306	13	0.995	0.993	0.643	0.927	11.982	99.427	1.543
	CARS200	106	17	0.998	0.993	0.323	0.922	12.041	176.317	1.432
	BIPLS-CARS	115	12	0.996	0.993	0.629	0.912	12.182	228.093	1.376
	BIPLS-GSA	138	15	0.996	0.993	0.597	0.904	12.283	1801.827	1.454
	CARS-GSA	70	12	0.998	0.993	0.438	0.893	12.435	1124.644	1.416

^1^ Number of wavelengths; ^2^ modeling time spent on selecting wavelengths and training the model; ^3^ testing time for predicting 30 new samples using the established model.

## Data Availability

The data presented in this study are available on request from the corresponding author.
